# Prognosis and immunoinfiltration analysis of angiogene-related genes in grade 4 diffuse gliomas

**DOI:** 10.18632/aging.205054

**Published:** 2023-09-21

**Authors:** Hui Liu, Zhirui Zeng, Peng Sun

**Affiliations:** 1 Department of Neurosurgery, Affiliated Hospital of Qingdao University, Qingdao, China; 2 Department of Neurosurgery, Guizhou Medical University, Guiyang, China

**Keywords:** angiogenesis-related, grade 4 diffuse gliomas, prognosis, immune infiltration

## Abstract

Although angiogenesis critically influences the progression of solid tumors, its contribution to highly malignant, grade 4 diffuse gliomas remains unclear. After analyzing 506 angiogenesis-related genes differentially expressed in grade 4 diffuse gliomas via LASSO and univariate and multivariate COX regression analyses, we constructed a nomogram based on COL22A1, IGFBP2, and MPO that accurately predicted patient survival. The nomogram’s performance was validated in an external patient cohort, and a risk score based on the formula COL22A1*0.148+IGFBP2*0.234+MPO*0.145 was used to distinguish high-risk from low-risk patients. Based on differentially expressed genes among risk groups, functional enrichment and drug sensitivity analyses were conducted, and the association between COL22A1, IGFBP2, and MPO expression and infiltrating immune cells and immune checkpoint genes was investigated. We next focused on COL22A1, and verified its overexpression in both glioma cell lines and clinical samples. A pro-oncogenic role for COL22A1, evidenced by impaired proliferation, migration, and invasion capacities, was evidenced upon shRNA-mediated COL22A1 silencing in glioma U87 and LN18 cells. In summary, we present a novel nomogram based on the angiogenesis-related genes COL22A1, IGFBP2, and MPO that allows survival prediction in patients with grade 4 diffuse gliomas. Furthermore, our cellular assays support a pro-oncogenic role for COL22A1 in these tumors.

## INTRODUCTION

In 2021, the World Health Organization (WHO) reclassified isocitrate dehydrogenase (IDH) wild-type glioblastoma (GBM) and grade 4 IDH mutant astrocytoma as grade 4 diffuse gliomas [1]. These neoplasias are characterized by a highly malignant nature, limited resectability, aggressive progression, and poor prognosis. Patients often present with symptoms indicative of progressive intracranial pressure, including headache and focal or evolving neurological deficits. Seizures are present in at least 25% of patients and may manifest later in the course of the disease in up to 50% of patients [2].

The current standard of care for GBM includes maximal safe surgical resection followed by concurrent radiation therapy and temozolomide (TMZ; an oral alkylating chemotherapy agent). Adjuvant chemotherapy with TMZ is then administered according to the National Comprehensive Cancer Network guidelines (NCCN, 2015) [3]. Unfortunately, due to the aggressive nature of the tumor, which determines its invasion into functional areas of the brain that govern language, motor function, and sensation, surgical resection of grade 4 diffuse gliomas is challenging and complete removal is not possible. Thus, radical resection of the primary tumor is insufficient for a cure, as remaining tumor cells in the surrounding brain tissue determine, in most patients, subsequent disease progression or recurrence [4].

Angiogenesis denotes the process that determines the formation of blood vessels. Inhibition of pathological angiogenesis has long been relevant in the field of oncology, and many recent advances have been made in angiogenesis research in oncology and other non-malignant or chronic conditions involving ophthalmological, cardiological, and gynecological disorders. In addition, angiogenic stimulants are used for ischemic disease, while angiogenic inhibitors are used to limit angiogenesis and modify or reprogram the vascular system as to further enhance immunotherapy [5].

Bioinformatics has prompted the identification of angiogenic genes in various tumor types such as stomach cancer [6], breast cancer [7], liver cancer [8], and colorectal cancer [9]. However, a thorough characterization of the angiogenesis-related gene landscape in grade 4 diffuse gliomas is lacking.

Studies investigating the mechanisms underlying the pathogenesis of advance-grade gliomas have pointed out the contribution of ferroptosis [10], necroptosis [11], pyroptosis [12], autophagy [13], and metabolic reprogramming [14], among other processes. However, little is currently known about the impact of angiogenesis-related genes on the onset and progression of grade 4 diffuse gliomas.

To address this knowledge gap, we analyzed data downloaded from public databases to explore, for the first time, the association between angiogenesis-related genes and grade 4 diffuse gliomas. The present findings offer a novel prognostic tool and may aid the development of novel therapeutic approaches to improve patient prognosis.

## RESULTS

### Identification of angiogenesis-related genes in grade 4 diffuse gliomas

To identify genes related to angiogenesis in grade 4 diffuse gliomas, we performed an intersection analysis of differentially expressed genes (DEGs) between glioma samples and normal adjacent brain tissue in the TCGA database, and known angiogenesis-related genes retrieved from the GeneCards and MSigDB repositories. The TCGA database initially included data from 173 patients. Of these, 6 patients with missing survival data were excluded, resulting in a final dataset of 167 patients. As a result, we obtained a list of 506 angiogenesis-related genes specific to grade 4 diffuse gliomas (Supplementary Table 1). Subsequently, using these 506 genes, we established a prognostic model for survival prediction, which was subsequently validated against an external cohort of Chinese glioma patients.

### Model establishment

**Figure 1:**
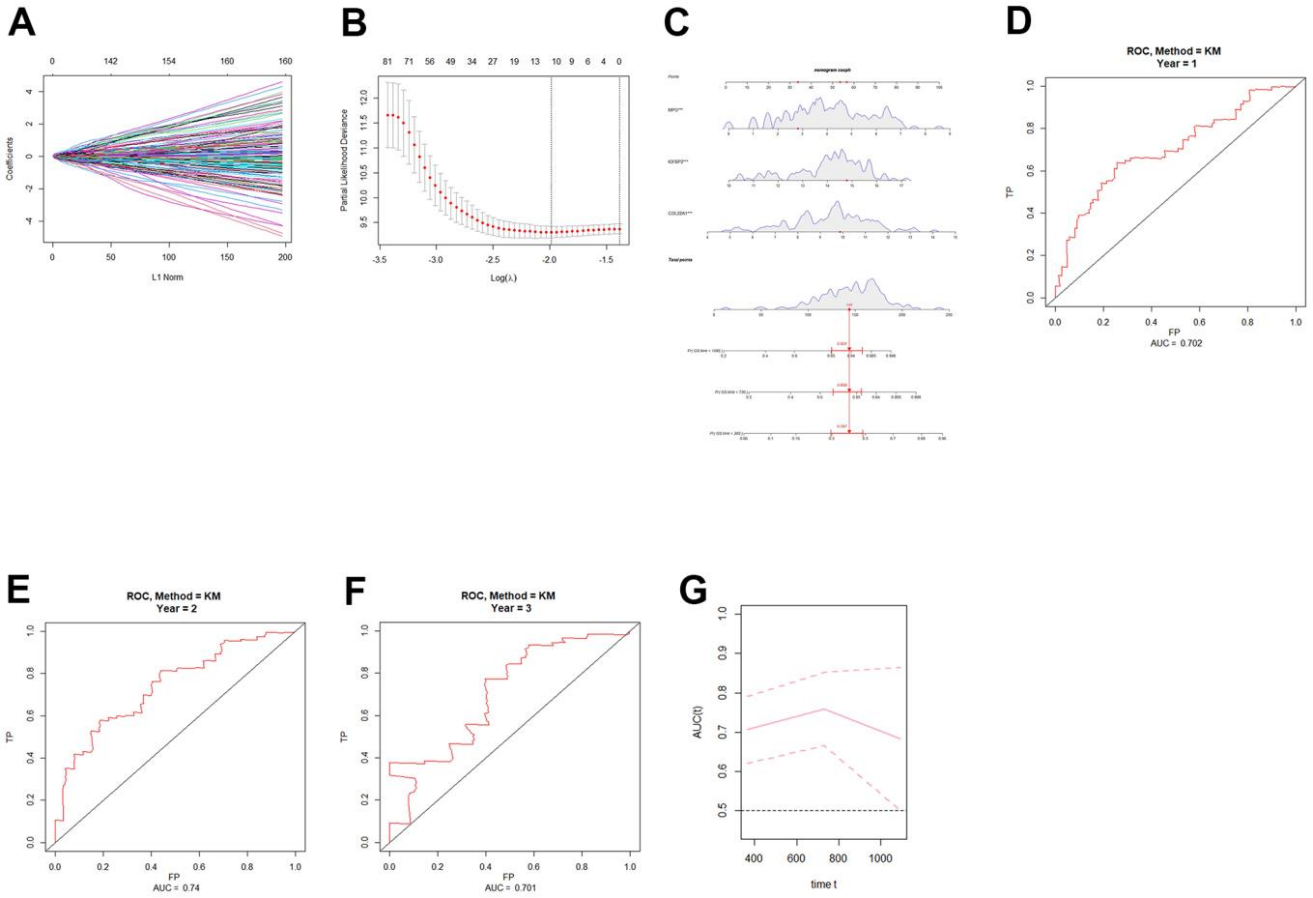
**Construction and validation of an angiogenic prognostic model for grade 4 diffuse gliomas.** (**A**, **B**) Lasso regression was used to screen angiogenesis-related prognostic genes in grade 4 diffuse gliomas. (**C**) Nomogram based on independent angiogenesis-related prognostic factors (COL22A1, IGFBP2, and MPO). (**D**–**F**) ROC curves for prediction of 1-year, 2-year, and 3-year OS by angiogenesis-related prognostic genes in grade 4 diffuse gliomas in TCGA database. (**G**) Time-dependent ROC-AUC with 95% confidence interval for 1-year, 2-year, and 3-year OS. X-axis time unit is day.

After the intersection analysis mentioned above, we identified 11 angiogenesis-related genes by employing LASSO Cox regression analysis. Subsequently, univariate COX regression was conducted to assess the association between these genes and patient prognosis (Table 1). Genes with a significance level of P < 0.05 were selected for further analysis using multivariate COX regression (Table 2). As a result, three genes, namely collagen XXII (COL22A1), insulin-like growth factor-binding protein 2 (IGFBP2), and myeloperoxidase (MPO), were identified as significant predictors (P < 0.05). Based on these genes, we constructed a nomogram (Figure 1C), and ROC curves were generated to evaluate its performance for 1-year, 2-year, and 3-year overall survival (OS) in the TCGA cohort. The AUCs for 1-year, 2-year, and 3-year were respectively 0.702, 0.74, and 0.701 (Figure 1D–1G). Finally, risk scores for grade 4 diffuse glioma patients in TCGA were calculated using the model (Supplementary Table 2). A set of DEGs of high-low risk group is shown in Supplementary Table 3.

**Table 1:** Results of the univariate COX proportional hazards analysis (HR, 95% confidence interval).

**Variables**	**Number of patients**	**HR (95% CI)**	***p*-value**
AR	167	0.92 (0.82-1.032)	0.154
COL22A1	167	1.2 (1.083-1.328)	<0.01
HOXB9	167	1.179 (1.072-1.296)	0.001
IGFBP2	167	1.205 (1.071-1.356)	0.002
MDK	167	1.355 (1.141-1.610)	0.001
MPO	167	1.178 (1.056-1.315)	0.003
NRG1	167	1.143 (1.041-1.255)	0.005
NRXN3	167	1.140 (1.030-1.261)	0.011
RETN	167	1.199 (1.086-1.322)	<0.01
SH2D2A	167	1.335 (1.150-1.550)	<0.01
TIMP1	167	1.255 (1.106-1.423)	<0.01

**Table 2:** Results of the multivariate analysis of various factors (HR, 95% confidence interval).

**Variables**	**Number of patients**	**HR (95% CI)**	***p*-value**
COL22A1	167	1.16 (1.04-1.293)	0.008
HOXB9	167	1.057 (0.951-1.176)	0.303
IGFBP2	167	1.264 (1.039-1.537)	0.019
MDK	167	1.001 (0.771-1.298)	0.995
MPO	167	1.156 (1.020-1.310)	0.023
NRG1	167	1.096 (0.977-1.228)	0.118
NRXN3	167	1.069 (0.941-1.213)	0.304
RETN	167	1.130 (0.996-1.281)	0.057
SH2D2A	167	0.976 (0.786-1.213)	0.828
TIMP1	167	1.000 (0.823-1.216)	0.998

### Model validation

**Figure 2:**
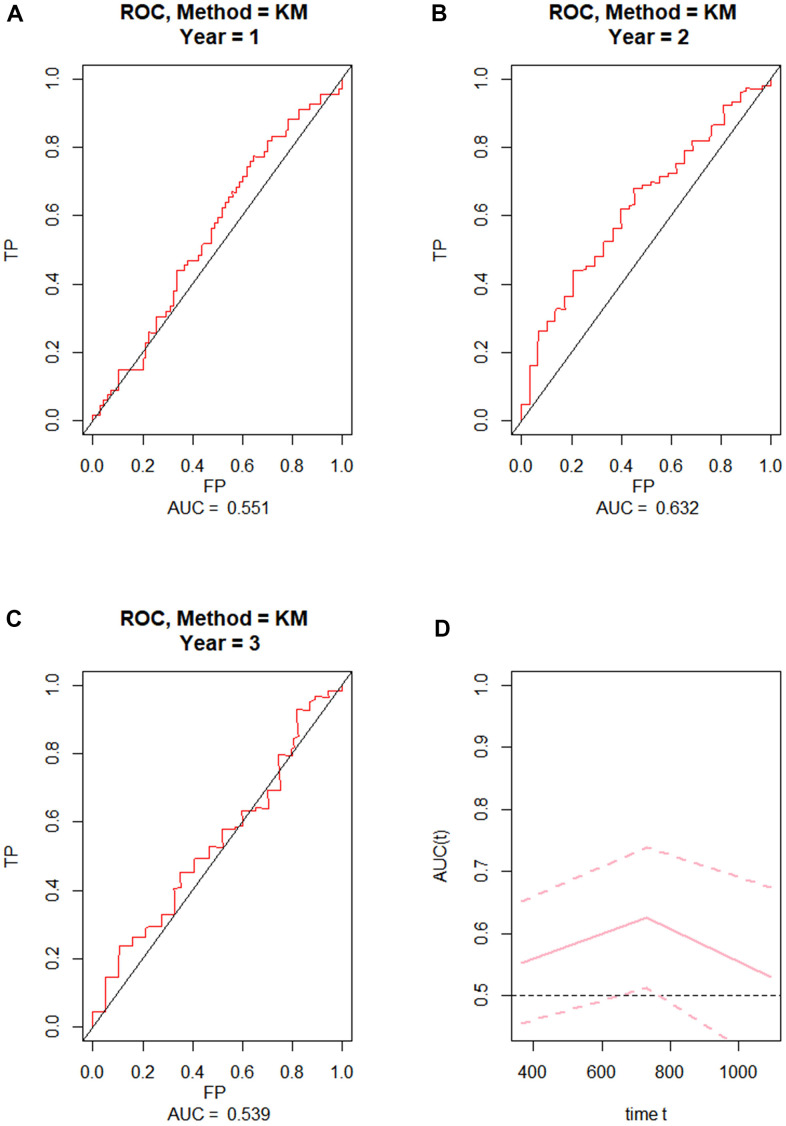
**External validation of the prognostic nomogram.** (**A**–**C**) ROC curves for predicting 1-year, 2-year, and 3-year OS in the CGGA dataset. (**D**) Time-dependent ROC-AUC with 95% confidence interval for 1-year, 2-year, and 3-year OS in the CGGA dataset. X-axis time unit is day.

To externally validate the clinical prediction model established using the TCGA dataset, ROC curve analysis of OS was performed on grade 4 diffuse glioma data from the Chinese Glioma Genome Atlas (CGGA) database (Figure 2A–2C). After applying the parameters of the prognostic model, the AUCs for 1-year, 2-year, and 3-year OS were respectively 0.551, 0.632, and 0.539. These results indicated that the prognostic model exhibited high reliability, especially for 2-year OS.

### Risk stratification and functional enrichment analysis

**Figure 3:**
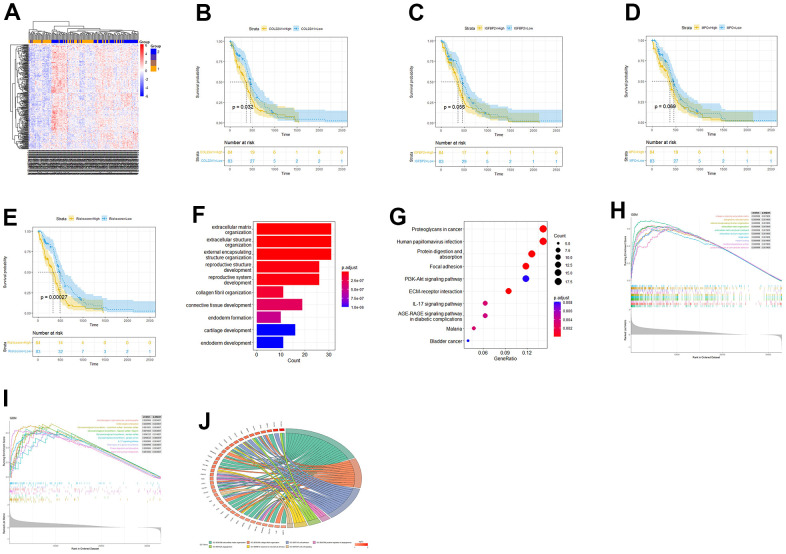
**Risk score-based OS and functional enrichment analyses.** (**A**) Heatmap of DEGs based on risk score stratification. (**B**–**E**) Kaplan-Meier curves based on COL22A1, IGFBP2, MPO, and risk scores. (**F**) Barplot of GO-BP terms enriched with DEGs between high- and low-risk groups. (**G**) Dot-plot of DEGs-enriched KEGG pathways. (**H**) GSEA based on GO. (**I**) GSEA based on KEGG. (**J**) Circos plot based on enriched GO terms.

The samples were then grouped based on their risk scores, and a heatmap was generated to visualize gene expression patterns (Figure 3A). Kaplan-Meier analysis was performed to assess the prognostic significance of COL22A1, IGFBP2, MPO gene expression and high-low risk groups of patients (Figure 3B–3E). The *p*-values of COL22A1, IGFBP2, MPO, and risk scores were respectively 0.032, 0.056, 0.069, and 0.00027. The hazard ratio (HR) for the high-risk vs the low-risk group was 1.88 (95% CI, 1.535-2.225). The analysis revealed that the survival rates of high risk groups were lower than those of low risk groups (P < 0.05).

Next, we conducted GO- and KEGG-based functional enrichment analyses of the DEGs (LogFC > 0 and P < 0.05) detected between the high- and low-risk groups. (Figure 3F, 3G). Upon GO analysis of biological processes (BP), the most enriched terms included extracellular matrix organization, extracellular structure organization, and collagen fibril organization (Figure 3F). On KEGG analysis, the most enriched pathways included protein digestion and absorption, focal adhesion, and ECM-receptor interaction (Figure 3G). To further investigate the potential involvement of the risk-related DEGs in specific BPs and pathways, we conducted Gene Set Enrichment Analysis (GSEA) for enriched GO and KEGG items and generated a Circos plot based on GO enrichment data. Upon GSEA based on GO analysis, the term extracellular matrix organization was still enriched (Figure 3H). Upon GSEA based on KEGG analysis (Figure 3I), the ECM-receptor interaction pathway was still enriched. A Circos plot showing the association between DEGs and enriched GO processes, including angiogenesis-related terms, is presented in Figure 3J.

### Analysis of immune cell infiltration

**Figure 4:**
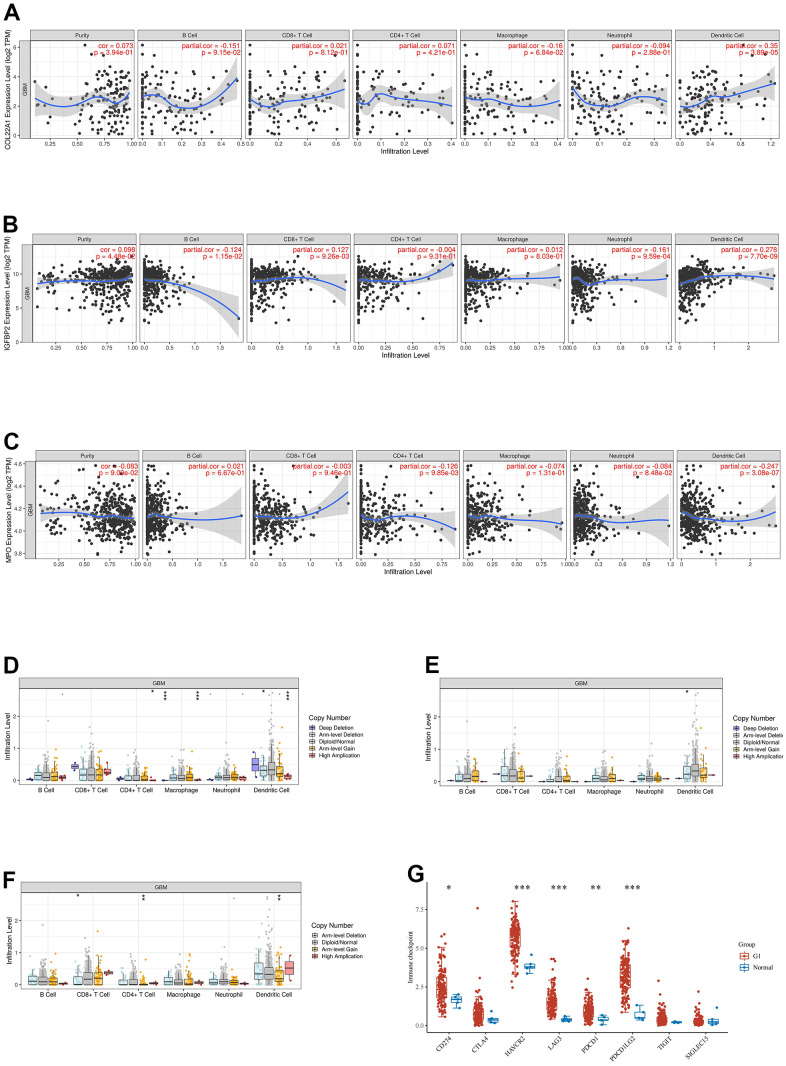
**Analysis of immune cell infiltration, SCNA, and immune checkpoint expression in grade 4 diffuse gliomas.** (**A**–**C**) Analysis of immune cell infiltration associated with COL22A1, IGFBP2, and MPO expression levels in grade 4 diffuse gliomas. (**D**–**F**) Association of SCNAs of COL22A1 (**D**), IGFBP2 (**E**), and MPO (**F**) with immune cell infiltration in grade 4 diffuse gliomas. (**G**) Immune checkpoint analysis of grade 4 diffuse gliomas vs normal brain.

The association between COL22A1, IGFBP2, and MPO expression and infiltrating immune cells in grade 4 diffuse gliomas was explored using the website https://www.aclbi.com/static/index.html#/immunoassay. Immune cell infiltration analyses (Figure 4A–4C) indicated positive correlations between COL22A1 expression levels and dendritic cells (DCs; Figure 4A), between IGFBP2 levels and B cells, CD8+ T cells, neutrophils, and DCs (Figure 4B), and between MPO levels and CD4+ T cells and DCs (Figure 4C). Meanwhile, associations with various immune infiltrating cells were observed for somatic copy number alterations (SCNAs) of COL22A1 (Figure 4D), IGFBP2 (Figure 4E), and MPO (Figure 4F). In turn, results of immune checkpoint analysis (Figure 4G) indicated overexpression of CD274, HAVCR2, LAG3, PDCD1 and PDCD1LG2 in grade 4 diffuse gliomas compared to normal brain.

### Drug sensitivity analysis

**Figure 5:**
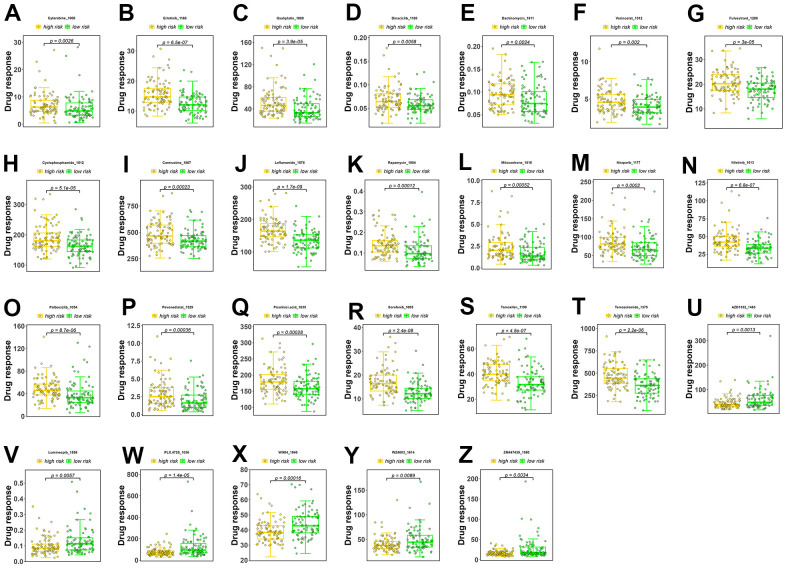
**Drug sensitivity analysis.** (**A**–**T**) Drugs with higher sensitivity in the low-risk group. (**U**–**Z**) Drugs with higher sensitivity in the high-risk group. The Y axis is IC50.

A sensitivity analysis was performed on 198 drugs, and box plots of drug sensitivity were generated for the high- and low-risk groups. Among the drugs analyzed, cytarabine, erlotinib, oxaliplatin, dinaciclib, dactinomycin, vorinostat, fulvestrant, cyclophosphamide, carmustine, leflunomide, rapamycin, mitoxantrone, niraparib, nilotinib, palbociclib, pevonedistat, picolinic acid, sorafenib, tamoxifen, and temozolomide exhibited higher sensitivity in the low-risk group (Figure 5A–5T). In contrast, AZD1332, luminespib, PLX.4720, WIKI4, WZ4003, and ZM447439 exhibited higher sensitivity in the high-risk group (Figure 5U–5Z).

### Prediction of patient response to immunotherapy

**Figure 6:**
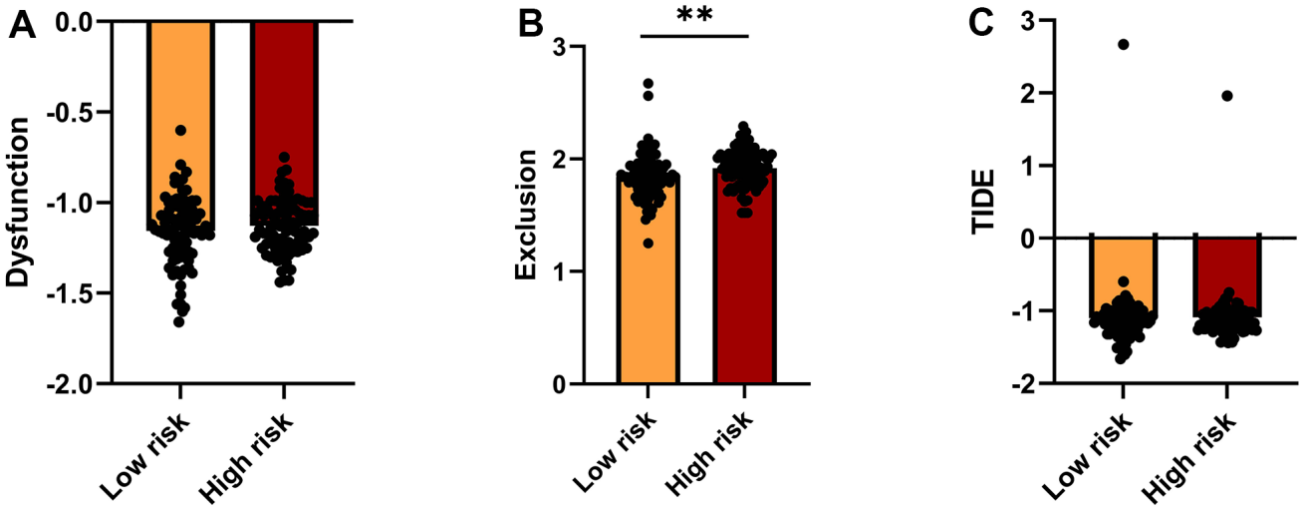
**TIDE analysis of immunotherapy response.** (**A**–**C**) TIDE-based Dysfunction scores (**A**), Exclusion scores (**B**), and overall TIDE scores (**C**) for the high- and low-risk groups.

The Tumor Immune Dysfunction and Exclusion (TIDE, http://tide.dfci.harvard.edu/) platform, which integrates large-scale omics data from numerous tumor cohorts, was accessed to predict immunotherapeutic response in low- and high-risk patients. T cell dysfunction, T cell exclusion, and TIDE scores for the two risk groups are shown in Figure 6A–6C. Compared to the low-risk group, higher exclusion scores in the high-risk group are indicative of lower immune cell infiltration.

### COL22A1 is a novel pro-oncogenic factor in grade 4 diffuse gliomas

**Figure 7:**
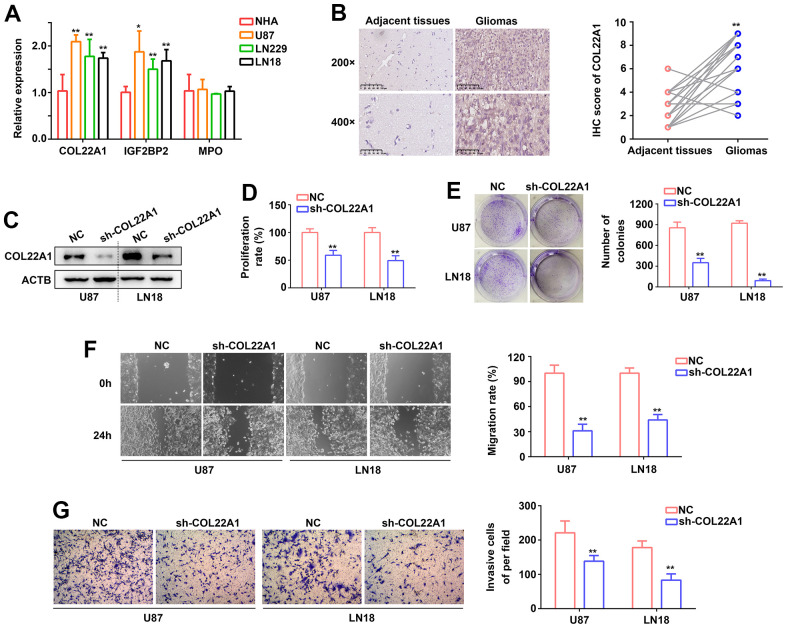
**COL22A1 knockdown inhibits glioma cell proliferation and migration.** (**A**) RT-qPCR was used to detect the expression of COL22A1, IGF2BP2, and MPO in NHA, U87, LN229, and LN18 cells. (**B**) IHC was used to detect the expression of COL22A1 in grade 4 diffuse gliomas and adjacent non-tumor tissues. (**C**) Western blotting was used to detect the expression of COL22A1 in negative control (NC) and COL22A1 knockdown cells. (**D**) The CCK-8 assay was used to measure proliferation in NC and COL22A1 knockdown cells. (**E**) Colony formation assays were used to detect tumorigenic potential of NC and COL22A1 knockdown cells. (**F**) Wound healing assays were used to assess migration rates in NC and COL22A1 knockdown cells. (**G**) Transwell assays were used to estimate invasion rates in NC and COL22A1 knockdown cells. *, P<0.05; **, P<0.01.

To define molecular assays assessing the impact of COL22A1, IGFBP2, and MPO dysregulation in glioma behavior, RT-qPCR was first used to determine the expression of the corresponding mRNAs in normal human astrocytes (NHAs) and three grade 4 diffuse glioma cell lines (U87, LN229, and LN18). Compared with NHAs, COL22A1, and IGF2BP2 mRNA levels were significantly increased in the glioma cell lines, while MPO levels were instead similar (Figure 7A). To establish a clinical correlation of these findings, we examined the expression of COL22A1 in 26 grade 4 diffuse glioma samples and normal adjacent tissues via IHC. Results demonstrated higher levels of COL22A1 in tumor specimens (Figure 7B).

Since the pro-tumorigenic effects of IGF2BP2 in grade 4 diffuse glioma are well-established, we were particularly intrigued by the potential pro-oncogenic role of COL22A1 in these tumors. Hence, we performed shRNA-mediated COL22A1 knockdown in U87 and LN18 cell. In both cells' lines, CCK-8 and colony formation assays revealed a decrease in proliferation and colony formation abilities (Figure 7C–7E), as well as significantly inhibited migration (Figure 7F) and invasion (Figure 7G) capacities, upon suppression of COL22A1. These results suggested that COL22A1 overexpression promotes the progression of grade 4 diffuse gliomas.

## DISCUSSION

In recent years, many studies have focused on the determinant influence of angiogenesis in cancer onset and progression [15]. Indeed, various angiogenesis-related genes had been proposed as independent prognostic factors in human cancers [16]. Hence, identifying and understanding the contribution of these genes to tumorigenesis provides a valuable foundation for developing effective therapeutic strategies to improve patient prognosis. However, few studies have assessed the relationship between angiogenesis-related genes and grade 4 diffuse gliomas. Therefore, in this study we investigated this association through bioinformatics methods, constructed a risk model based on three angiogenesis-related genes differentially expressed in grade 4 diffuse gliomas, and demonstrated a pro-oncogenic role for COL22A1 in malignant glioma cells *in vitro*.

The risk model proposed here, based on the COL22A1, IGFBP2*,* and MPO genes, yielded good performance in predicting the prognosis of patients. Survival analyses on TCGA samples revealed that grade 4 glioma patients in the low-risk group exhibited a higher survival rate and a more favorable prognosis. Importantly, we demonstrated acceptable prognostic ability for the model upon testing in an external cohort of Chinese patients.

Recent studies reported prognostic significance in diffuse glioma for ITGA3 and MAP1LC3A, two autophagy-related genes [13], BCL7A, a protein involved in ATP-dependent chromatin remodeling [17], and lipid metabolism genes [18]. More recently, Yuan et al. established a positive association between M^6^A methylation status and glioblastoma stemness, immune checkpoint expression, and promotion of an immunosuppressive environment [19]. Our current study, which focused exclusively on grade 4 diffuse gliomas, the most aggressive primary brain tumors, revealed that a nomogram based on COL22A1, IGFBP2, and MPO expression can reliably evaluate patient prognosis. To our knowledge, no prognostic models related to these genes have hitherto been reported.

It has been established that COL22A1 maintains vascular stability and may be associated with intracranial aneurysms [20]. Early reports demonstrated that IGFBP2 is overexpressed in high grade gliomas [21, 22]. More recently, a study revealed that IGFBP2 promotes vasculogenic mimicry by modulating CD144 and MMP2 expression in glioma [23]. Interestingly, IGFBP2 expression was shown to promote neural stem cell maintenance and proliferation, and was associated with differences in glioblastoma subtypes [24]. In a recent meta-analysis, IGFBP2 expression was associated with poor prognosis in several tumors, including glioma, suggesting its potential as a prognostic biomarker in cancer patients [25]. MPO is a major component of activated neutrophils and macrophages and a key mediator of the innate immune system [26]. Plasma levels of MPO are typically elevated in patients with acute coronary syndromes [27], and its expression has been positively associated with atherosclerosis progression [28]. Interestingly, animal experiments showed that MPO exhibited antitumor activity against glioma after radiotherapy [29].

Cancer prognosis is impacted by the infiltration of immune cells into the tumor microenvironment [30]. It is widely acknowledged that immunotherapies such as chimeric antigen receptor (CAR) T cell therapy can selectively target and kill cancer cells. Natural killer (NK) cells play a dual role by controlling both the growth of GBM cells and mediating the suppression of tumor progression [31]. DCs contribute to immune recognition of GBM and synergize with other immune cells in the context of radiotherapy and chemotherapy [31]. CD4^+^ Th1 cells, activated by CD8^+^ T cells, and γδ-T cells predominantly contribute to type I immune responses and are associated with good prognosis in lung cancer patients [32, 33]. Conversely, Th2 cells, Th17 cells, and Foxp3^+^ regulatory T (Treg) cells are commonly associated with tumor progression and poor prognosis [34]. In gastric carcinoma, the prognostic impact of immune cells such as Th2 cells, T helper cells, and mast cells has been observed [35].

Our GO and KEGG analysis showed that DEGs in the high-risk group were significantly enriched in several angiogenesis-related biological processes and pathways, including extracellular matrix composition, ECM-receptor interaction, focal adhesion, collagen fiber composition, and positive regulation of angiogenesis. Although an association between collagen COL22A1 expression and vascular stability has been reported [20], the oncogenic role of COL22A1 in relation to tumor angiogenesis remains uncertain. Based on the novel evidence herein reported, we propose that COL22A1 is a positive modulator of angiogenesis in grade 4 diffuse gliomas and hence represents a plausible therapeutic target.

However, further research is necessary to overcome some limitations of this study. Although RT-qPCR, IHC, western blotting, and cell proliferation and migration assays indicated a clear pro-oncogenic role for COL22A1, the specific mechanisms involved remain undefined. Likewise, further research is needed to assess the potential pro-angiogenic actions of MPO in advanced glioma. Also, there is always the possibility of supervisor error or selection bias when screening data from databases.

In summary, the present study identified angiogenesis-related genes associated with prognosis and immune cell infiltration in patients with grade 4 diffuse gliomas, thus refining the current understanding of this disease. The novel nomogram hereby presented, based on COL22A1, IGFBP2 and MPO mRNA expression levels, can accurately predict the survival of patients. Moreover, based on our *in vitro* experiments, we propose that COL22A1 is a novel pro-oncogenic factor in grade 4 diffuse gliomas.

## MATERIALS AND METHODS

### Data collection and differential gene expression screening

RNA sequencing expression profiles of grade 4 diffuse gliomas (n=173) were obtained from The Cancer Genome Atlas (TCGA) database (https://portal.gdc.cancer.gov/projects/TCGA-SKCM) and accessed from the UCSC Xena website (https://xenabrowser.net/) lastly. Complete survival data were available for all but six patients. For the identification of angiogenesis-related genes, we sourced relevant gene lists from two reliable databases, namely GeneCards, from which we obtained a list of 1138 genes (Supplementary Table 4), and the Molecular Signatures Database (MSigDB) (http://www.broad.mit.edu/gsea/msigdb), from which we extracted 8 curated gene sets (Supplementary Table 5).

To identify prognostic target genes, we conducted differential expression analysis comparing glioma samples and matched paracancerous tissues retrieved from TCGA. We utilized the R package “limma” to identify differentially expressed (LogFC >1, P < 0.05) genes specific to grade 4 diffuse gliomas.

### Identification of angiogenesis-related genes in grade 4 diffuse gliomas

DEGs, screened from TCGA, and angiogenesis-related genes obtained from the MSigDB database and GeneCards were intersected to obtain differentially expressed angiogenesis-related genes in patients with grade 4 diffuse gliomas.

### Establishment of a prognostic model based on differentially expressed, angiogenesis-related genes

A prognosis model for grade 4 diffuse gliomas based on differentially expressed, angiogenesis-related genes was subsequently established and verified experimentally.

First, the screened glioma-related angiogenesis-related genes and patients’ clinical features were analyzed by univariate Cox regression, and significant factors (P < 0.05) were then included in Cox multivariate analysis. Factors with a P < 0.05 on were thus obtained and used to establish a nomogram. To verify the model’s accuracy, ROC curves were generated to predict the 1-year, 2-year, and 3-year overall survival (OS). Furthermore, Kaplan-Meier curves were conducted for the identified risk factors.

### Prognostic model validation

Upon establishment of the clinical prediction model, external validation was conducted based on RNA-seq data of 137 patients with grade 4 diffuse gliomas collected from the Chinese Glioma Genome Atlas (CGGA) database (http://www.cgga.org.cn/). Model accuracy was assessed by generating ROC curves for 1-year, 2-year, and 3-year OS.

### Risk stratification

For risk stratification of glioma patients, risk scores were calculated with the formula: Risk score = COL22A1*0.148+IGFBP2*0.234+MPO*0.145. Subsequently, the risk scores were used to categorize samples into high- and low-risk groups. Further analysis was performed on the DEGs, and heatmaps were generated to visualize the results. Additionally, Kaplan-Meier curve analysis was conducted to evaluate the prognostic value of the risk scores.

### Functional enrichment analysis

To gain insight into the biological functions and pathways associated with DEGs between the high- and low-risk groups, functional enrichment analyses were conducted based on Kyoto Encyclopedia of Genes and Genomes (KEGG) and Gene Ontology (GO) databases. Gene Set Enrichment Analysis (GSEA) diagrams and enrichment plots were then generated to visually present the pathways and highlight their significance in grade 4 diffuse gliomas.

### Drug sensitivity analysis

Drug sensitivity analysis was conducted for the three identified risk factors, namely COL22A1, IGFBP2 and MPO. We utilized the “oncoPredict” R package, which leverages Genomics of Drug Sensitivity in Cancer (GDSC) data to assess drug sensitivity. A total of 198 drugs were subjected to sensitivity analysis. The results were then used to design box and whisker plots illustrating drug sensitivity in high-risk and low-risk patients.

### Immune checkpoint analysis

We utilized the website https://www.aclbi.com/static/index.html#/immunoassay to conduct immune checkpoint analysis and collected data on immune cell infiltration for COL22A1, IGFBP2 and MPO genes from TIMER 2.0 (http://timer.comp-genomics.org/). We also analyzed somatic copy number alteration (SCNA) data for these genes and identified immune checkpoints associated with grade 4 diffuse gliomas.

### Prediction of patient response to immune checkpoint blockade therapy

The expression profiles of therapy-naïve grade 4 diffuse glioma samples were analyzed with the Tumor Immune Dysfunction and Exclusion (TIDE, http://tide.dfci.harvard.edu/) algorithm. Upon cross-referencing multiple published transcriptomic biomarkers, scores are derived for T cell exclusion and T cell dysfunction/exhaustion status for each tumor profile. The overall TIDE score predicts the susceptibility of the tumor to immune checkpoint blockade.

### Immunohistochemical (IHC) staining

A total of 26 grade 4 diffuse glioma samples from patients with no history of radio-chemotherapy or targeted therapy were collected from the Affiliated Hospital of Qingdao University with the approval of the Institutional Ethics Committee. All patients provided written informed consent. IHC was performed as previously described by Zeng et al. [36] using an anti-COL22A1 antibody (PA5-55200, Thermo Fisher Scientific, Waltham, MA, USA).

### Cell culture and shRNA transfection

Normal human astrocytes (NHA) and three glioma cell lines, including U87, LN229, and LN18, were obtained from ATCC (Manassas, VA, USA). All cells were cultured in RPMI-1640 medium (Beyotime, Shandong, China) with 10% FBS (HyClone, Thermo Fisher Scientific, USA). Negative control (NC) and COL22A1-targeted shRNAs were obtained from iGeneBio (Beijing, China) and sub-cloned into the pLV-Puro vector. After transfection of shRNAs using polybrene reagent (iGeneBio), the cells were incubated for 48 h and then treated with 0.5 μg/ml puromycin to select stable knockdown cells.

### RT-qPCR

Total cellular RNA was extracted using an EZ-press RNA Purification Kit (HiFunBio, Shandong, China) according to the manufacturer’s instructions. After determining RNA concentrations in samples using a fluorescence spectrometer, reverse transcription of 800 ng mRNA was performed using a First Strand cDNA Synthesis Kit (Qiagen, Germany). TB green Premix Ex Taq II kit (Tli RNase H Plus; Takara, Japan) to assess via RT-qPCR the expression of target genes, including COL22A1, IGF2BP2, MPO, and ACTB, with relative expression levels normalized to ACTB. The following primers were used: COL22A1 (forward: 5’-TCCGACTTCAATGCCATCGAC-3’; reverse: 5’- TACACGAACGCTAGGACAGAG-3’); IGF2BP2 (forward: 5’-AGTGGAATTGCATGGGAAAATCA-3’; reverse: 5’-CAACGGCGGTTTCTGTGTC-3’); MPO (forward: 5’-TGCTGCCCTTTGACAACCTG-3’; reverse: 5’-TGCTCCCGAAGTAAGAGGGT -3’); and ACTB (forward: 5’-CATGTACGTTGCTATCCAGGC-3’; reverse: 5’-CTCCTTAATGTCACGCACGAT-3’).

### Western blotting

Protein extraction from U87 and LN18 cells was performed using RIPA buffer (Absin, Shandong, China) supplemented with 1% PMSF (Absin). A total of 30 μg protein from each cell group was loaded per lane and subjected to electrophoresis on a 12% SDS-PAGE gels (Absin), transferred into PVDF membranes (Invitrogen, USA) and blocked with TBST containing 5% milk powder for 30 min. The membranes were then incubated with COL22A1 (PA5-70815, Thermo Fisher Scientific, USA) and ACTB (PA5-78715, Thermo Fisher Scientific, USA) antibodies. After washing in TBST, HRP-conjugated secondary antibodies were applied, and protein bands visualized using an ECL reagent (Absin). The expression of COL22A1 was normalized to that of ACTB.

### Cell proliferation assay

U87 and LN18 cells were seeded in 96-well plates at a density of 2 × 10^3^ cells/well. After culturing for 48 h, 10 μL of CCK-8 reagent (Beyotime, Jiangsu, China) was added. After a 2-h incubation, proliferation rates were estimated based on absorbance detected at 450 nm.

### Colony formation assay

A total of 1.5 × 10^3^ U87 and LN18 cells/well were seeded into 6-well plates. After culturing for 12 days, the medium was removed and cells were fixed with 4% paraformaldehyde. Then, 1% crystal violet was used to stain cell colonies. The dye was removed by washing with PBS, and the number of cell colonies was photographed and counted.

### Cell migration and invasion assays

The effect of COL22A1 knockdown on cell migration and invasion capacity was examined in U87 and LN18 cells using wound healing and Transwell assays, respectively. For wound healing assay, cells were seeded in 6-well plates and cultured until they reached over 95% confluence. Subsequently, a wound was traced in the cell monolayer using a sterile pipette tip. The wells were then washed with PBS to remove any floating cells. The wounds were photographed at various time points, ranging from 0 to 24 h, to quantify the migratory ability of the cells based on wound closure. cells were suspended in RPMI-1640 medium lacking FBS and placed in the upper chambers (Corning, USA) that had been pre-coated with 8% matrigel (Corning, USA). The lower chambers (Corning, USA) contained DMEM medium with 10% FBS as the inducer. After 24 hours, the upper chamber was fixed with 4% paraformaldehyde and stained with 0.5% crystal violet. The average number of invasive cells was determined by counting in five randomly selected fields.

### Statistical analysis

Experimental results were analyzed using SPSS 20.0 software. Comparisons among two groups were performed using unpaired t-test. P < 0.05 was considered significant.

### Data availability

This article’s data were obtained from publicly available sources, including TCGA database (https://portal.gdc.cancer.gov/projects/TCGA-SKCM), Molecular Signatures Database (MSigDB, http://www.broad.mit.edu/gsea/msigdb), and CGGA database (http://www.cgga.org.cn/).

### Consent for publication

All authors provided consent for publication of the article.

## Supplementary Material

Supplementary Table 1

Supplementary Table 2

Supplementary Table 3

Supplementary Table 4

Supplementary Table 5
