# Supplementary material for: Prognosis and immunoinfiltration analysis of angiogene-related genes in grade 4 diffuse gliomas
Source: Aging (Albany NY). 2023 Sep 21;15(18):9842–57. doi: 10.18632/aging.205054 (PMC10564429; doi:10.18632/aging.205054)
Supplement: Supplementary Table 5 [file aging-15-205054-s005.pdf]

SUPPLEMENTARY TABLE

Supplementary Table 5. Angiogenesis-related genes from the molecular signatures database (MSigDB).

CANX
EMCN
EPGN
FOXO4
NPPB
SPINK5
THY1
TNNI3
